# Hair Shaft Fracture in a Young Athlete: A Rare Case Report of Acquired Trichorrhexis Nodosa

**DOI:** 10.7759/cureus.67341

**Published:** 2024-08-20

**Authors:** Anannya S, Sharada R G, Narasimhalu C R V, Sai Kavya D, Guntamukkala Geeta Sai

**Affiliations:** 1 Department of Dermatology, Saveetha Medical College and Hospital, Saveetha Institute of Medical and Technical Sciences (SIMATS), Saveetha University, Chennai, IND

**Keywords:** paintbrush fracture, scanning electron microscopy, trichoscopy, weathering, athlete, trichorrhexis nodosa

## Abstract

Trichorrhexis nodosa (TN) is a hair shaft anomaly characterized by abnormal fragility and weak points (nodes) in the hair shaft. It can be congenital or acquired, with the acquired form being more common. Patients typically present with lusterless and fragile hair, an inability to grow hair to a normal length, fraying at the distal free end, and white knots and fractures along the hair shaft. This condition is often linked to daily hair care routines and exposure to chemicals or environmental factors that cause trauma to the hair. Diagnosis is typically confirmed through dermoscopy or microscopy, which reveal characteristic paintbrush-like hair fractures. Hair anomalies in sports have been rarely documented. Here, we report a unique case of acquired distal TN in a 19-year-old male professional volleyball player and a recreational swimmer with exposure to multiple environmental factors such as years of chlorine water exposure and training that may have contributed to this condition. The patient presented with a three-month history of abnormal hair fragility and glistening white areas along the hair shafts. Trichoscopy revealed multiple white nodes along the hair shafts, while light microscopy displayed nodular swellings with disruption of the cuticle and splaying out of cortical fibers. Scanning electron microscopy (SEM) further demonstrated the classical "two brooms stuck in opposite direction" or "paintbrush fracture" appearance. Based on the clinical presentation and microscopic findings, a diagnosis of diffuse distal acquired TN was made. The patient was advised to tonsure his head and use gentle, sulfate-free shampoo for regrown hair. Follow-up at six months showed no recurrence of lesions, highlighting the importance of identifying and addressing potential causative factors in managing this condition.

## Introduction

Trichorrhexis nodosa (TN) is an uncommon hair shaft disorder where the focal defect in the hair fiber characterized by thickening or weak points (nodes) leads to abnormally fragile hair that breaks off easily [1]. This condition can manifest in two forms: inherited or acquired, with the latter being more prevalent in clinical practice [1]. While the exact incidence rates of inherited and acquired TN are not well-established in the literature, it remains a significant concern in dermatological practice.

The condition was first described in 1852 by Samuel Wilks of Guy's Hospital, marking the beginning of scientific inquiry into this unique hair disorder. Later, in 1876, Kaposi proposed the term "trichorrhexis nodosa," which has since become the standard nomenclature for this condition [2,3]. This historical context underscores the long-standing recognition of TN in the medical community.

Clinically, TN presents with distinct characteristics that aid in its identification. Patients typically report lusterless and fragile hair, often accompanied by an inability to grow hair to a normal length. Upon closer examination, fraying at the distal free end of the hair is commonly observed. Additionally, some hairs may exhibit white knots and fractures along the shaft, giving the hair a distinctive appearance.

The etiology of acquired TN is multifaceted, often linked to daily hair care practices, contact with chemicals or exposure to environmental factors that cause trauma to the hair [4]. These factors can include, but are not limited to, excessive heat styling, harsh chemical treatments, and frequent exposure to chlorinated water or saltwater. Understanding these potential triggers is crucial for both diagnosis and management of the condition.

Diagnostic approaches for TN have evolved over time, with modern techniques offering greater precision. Dermoscopy has emerged as a valuable tool in clinical practice, allowing for noninvasive examination of the hair shaft. This method often reveals paintbrush-like hair fractures, a hallmark sign of TN. Microscopy, both light and electron, provides even more detailed visualization of the hair shaft abnormalities, further confirming the diagnosis.

The significance of TN extends beyond its immediate cosmetic impact. For many patients, the condition can cause considerable distress, affecting self-esteem and quality of life. Moreover, TN can sometimes be associated with underlying systemic conditions or nutritional deficiencies, highlighting the importance of a thorough clinical evaluation.

Treatment strategies for TN are primarily focused on identifying and eliminating the causative factors. This may involve modifying hair care routines, avoiding certain chemical treatments, and protecting the hair from environmental stressors. In some cases, nutritional supplementation or treatment of underlying conditions may be necessary.

In this case report, we present a young male patient with acquired distal TN, whose condition developed in the context of multiple potential environmental triggers. He is a professional volleyball player with 10 years of training history and a recreational swimmer for the past eight years with chronic chlorine water exposure. This case is particularly instructive as it demonstrates the complex interplay of factors that can contribute to the development of TN in otherwise healthy individuals.

By examining this case in detail, from initial presentation through diagnosis and management, we aim to contribute to the growing body of knowledge on TN. This case report not only highlights the clinical features and diagnostic approach to TN but also emphasizes the importance of a comprehensive patient history in identifying potential causative factors. To the best of our knowledge, this case report represents one of the first documented instances of TN in a competitive athlete, shedding light on the potential interplay between sporting activities and hair shaft disorders, thereby adding to the scientific literature.

## Case presentation

**Figure 1:**
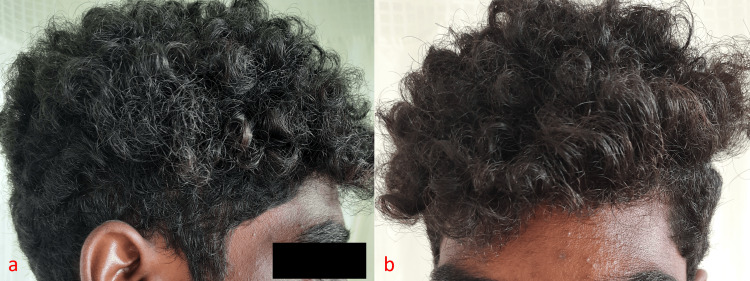
(a) Dull and brittle scalp hair; (b) dry and lusterless scalp hair

A 19-year-old male patient presented to the Dermatology outpatient clinic with abnormal fragility of the hair and glistening white areas for three months. The patient had no past medical or surgical history or atopic background. There were no similar cases in the family. The patient was a volleyball player, training for two hours a day with daily vigorous shampooing. The patient also reported a history of frequent swimming in chlorinated pools and sea bathing. Scalp examination revealed normal hair density, length, and caliber but multiple whitish nodes along the length of the hair shaft with brittleness and loss of luster of hair (Figure 1a and Figure 1b).

**Figure 2:**
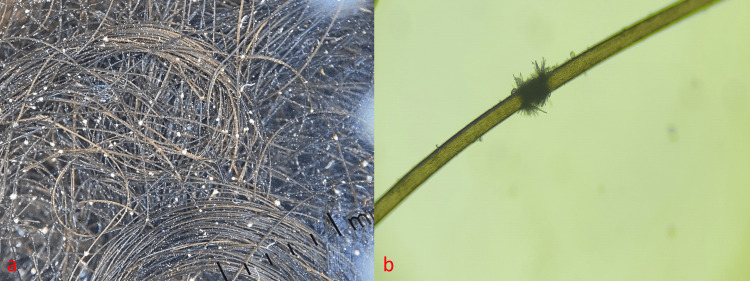
(a) Trichoscopy findings of multiple white nodes at the hair shafts (DermLite DL4); (b) light microscopy findings such as fraying of cortical fibers along the shaft, classical appearance of nodes along hair shaft combined with transverse hair shaft fractures producing thrust paintbrush appearance

Facial, body, and pubic hair were not involved. A diagnosis of TN was made after confirmation with dermoscopy and light microscopy. Trichoscopy showed multiple white nodes in hair shaft (Figure 2a). Light microscopy of a sample hair specimen revealed fraying of cortical fibers, "two brooms stuck in opposite direction" appearance and white nodes along the hair shafts (Figure 2b).

**Figure 3:**
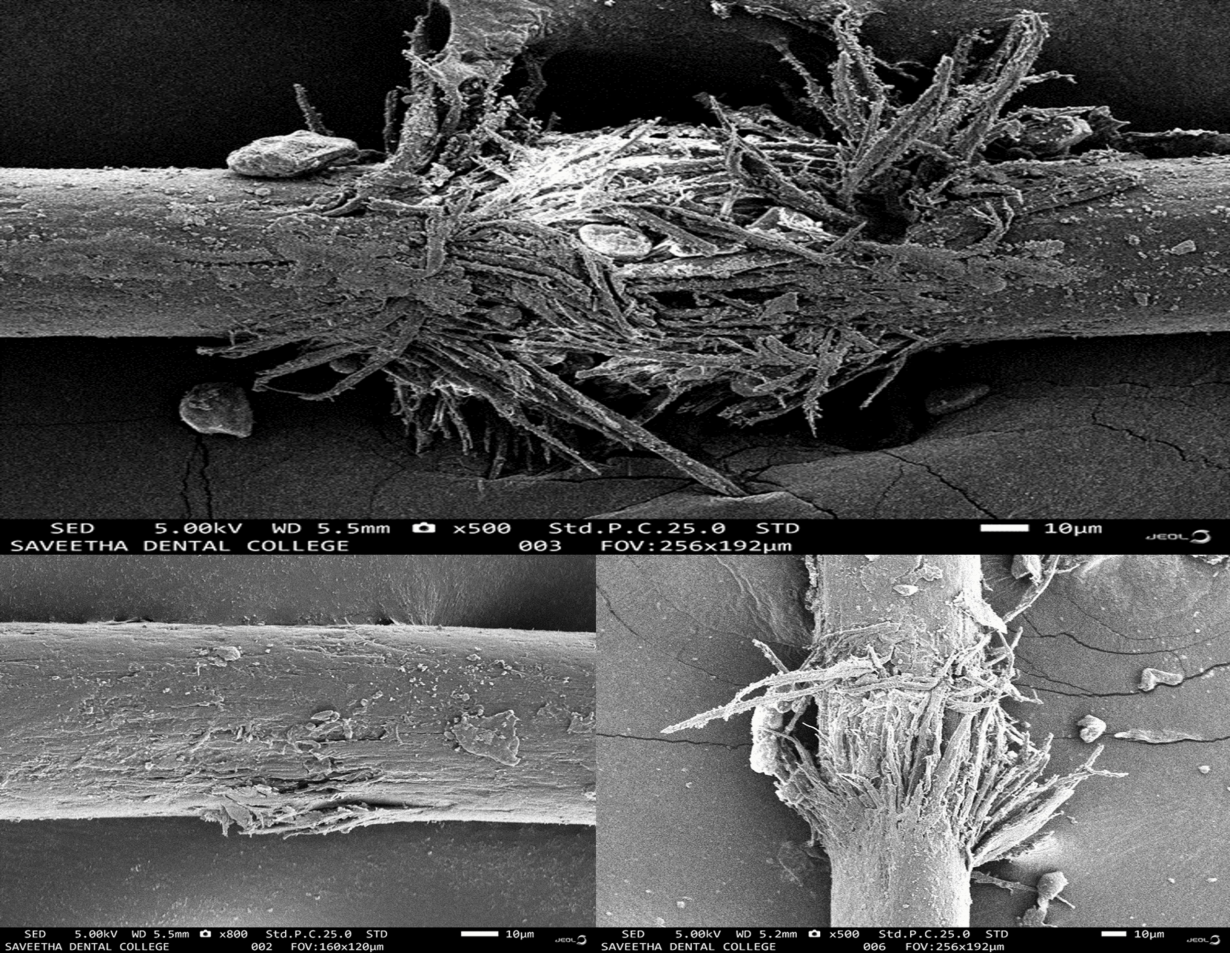
Under scanning electron microscope (SEM), the hair in the affected area resembles a crushed paintbrush, called “paintbrush fracture”

Scanning electron microscopy (SEM) of the hair specimen showed nodes resembling a crushed paintbrush (Figure 3).

Routine laboratory investigations were carried out and found to be normal. Other blood investigations to assess nutritional status, like levels of vitamin B12, vitamin D, and serum ferritin, were also found to be within normal limits. Cutting the hair or tonsuring the head was suggested to remove the damaged hair, and the patient was advised to do the following: usage of gentle, sulfate-free shampoo and leave-in conditioner, reduction of sunlight exposure, indoor sports training, avoidance of sea bathing, and application of natural oils like coconut oil. The patient was followed up after three months and after six months, and there was normal hair luster and length, without increased fragility or white nodes.

## Discussion

TN is an uncommon hair shaft disorder that occurs due to weathering. When a weak hair shaft partially fractures and the cortical cells fragment, uneven spurs form at the fracture point [5]. Samuel Wilks of Guy's Hospital initially reported this hair disorder in 1852, but it wasn't until 1876 that Kaposi coined the term TN [2,3]. Clinically, it presents with white flecking, dry, and lusterless hair with abnormal fragility and inability to attain normal hair length because of premature breakage of the hair fiber. The exact incidence rates of inherited and acquired TN are not well-established in the literature [3,6]. Over the course of a decade, 129 hair samples from 119 patients were evaluated retrospectively, and it was discovered that 13 individuals had TN, six cases of uncombable hair syndrome, and 25 patients of loose anagen hair syndrome [7]. Acquired form is more frequently reported in individuals with African or African-American hair types [3]. The incidence of TN specifically in sports settings is not well-documented in the literature. However, it is known to occur more frequently in individuals who subject their hair to repeated mechanical or chemical trauma, which can include certain sporting activities [8].

A primary congenital type, TN associated with other syndromes, and acquired TN are the three variations into which this disorder can be distinguished [9].

Acquired TN is more frequent in patients of African origin and skin of color, likely due to the structural properties of the hair shaft [10]. Causes include mainly physical factors like excessive or repeated trauma caused by blow-drying, ironing the hair, over-brushing, backcombing, stressed hairstyles, and scalp massage [4]. Notable behaviors that can cause enough harm include the following: rhythmic movement disorders like head-rolling and head-banging; habit disorders like hair pulling and tics; trichotillomania; and the scratching associated with pruritic dermatoses (like pediculosis capitis and seborrheic dermatitis). Among chemical injuries to the hair shaft, frequent swimming in chlorinated water, shampooing, excessive exposure to salt water, lengthy and repeated ultraviolet exposure, setting, perming, bleaching, and dyeing of the hair are mostly implicated [3,4]. There are three categories for acquired TN: proximal form, distal form, and localized form [3].

Acquired distal type affects mostly Asians and Whites. The whitish nodes are seen on the distal ends of the hair shaft, which is lusterless and shows splitting. Acquired proximal type is seen in Blacks who use excessive chemicals for styling hair with breakage in the proximal shaft, sometimes leading to alopecia. Localized TN is due to pruritic dermatosis over scalp, beard, or moustache, with repeated scratching or rubbing causing hair shaft breakage and faded hair in a small patch.

Congenital TN can present with concurrent symptoms of mental retardation, motor defects, growth failure, and seizures. Other associated symptoms may include nail and skin changes (ichthyosis), photosensitivity, ocular dystrophy, and infertility. A family history of the above conditions with similar hair problems may be present in such patients.

TN can be associated with hypothyroidism, argininosuccinic aciduria, iron deficiency, Menkes kinky hair syndrome, monilethrix, ectodermal dysplasia, trichothiodystrophy, biotin deficiency, and trichohepatoenteric syndrome [11,12].

Sometimes, other structural abnormalities of hair with increased fragility, including trichorrhexis invaginata, monilethrix, pili torti, and pseudomonilethrix, can later cause TN [13]. Rare causes can be tumor necrosis factor-α inhibitor therapy and P63 gene mutation.

Microscopy is considered the gold standard for its diagnosis. Classical appearance of nodes and transverse fractures in hair shaft producing “two brooms stuck in opposite direction” [14]. Clinical differentials include pediculosis, dandruff, and various abnormalities of the shaft such as pili annulati [1]. Trichoscopically, hair casts and trichorrhexis invaginata (bamboo hair) can be viewed as potential differential diagnosis [15]. The goal of treatment for acquired TN is to avoid the implicated offending agent. By lowering hair friction, the application of oils as well as conditioners can help prevent cuticular damage to the hair shaft [9].

The development of TN in this patient can be attributed to multiple factors. The mechanical stress from vigorous shampooing and friction during sports activities could have weakened the hair shaft. Prolonged exposure to chlorinated water and sea water may have altered the hair's chemical structure, making it more susceptible to damage. UV radiation from outdoor training could have further compromised the hair's integrity. The successful resolution of symptoms after eliminating these factors supports the hypothesis of their causal role. However, the possibility of genetic predisposition or other unidentified factors cannot be completely ruled out.

The strengths of this case report lie in its detailed clinical, trichoscopic, and microscopic findings of TN in an athlete with a long history of sports exposure. It highlights the potential cumulative effects of environmental factors on hair shaft health. The report also demonstrates successful management through simple interventions. The limitation is that as a single case report, it cannot establish a causal relationship between sports activities and TN. The lack of a control group or larger sample size limits generalizability. Additionally, other potential contributing factors such as genetic predisposition or nutritional status were not extensively explored.

## Conclusions

This case report of acquired diffuse TN in a young male with a 10-year history of volleyball training and eight years of swimming exposure highlights the potential long-term impact of sporting activities on hair health. The patient's extensive exposure to various environmental factors, including vigorous shampooing, frequent chlorinated pool and seawater contact, and excessive UV radiation, likely contributed to the development of this condition. This case underscores the importance of considering cumulative environmental exposures when evaluating athletes with hair shaft disorders. The successful management through simple interventions demonstrates the effectiveness of addressing these contributing factors. Furthermore, this report adds valuable insights to the limited literature on TN in athletes, particularly in the context of sporting activities including water sports and environmental exposures. By raising awareness of this condition, its varied etiology, and its association with long-term athletic pursuits, clinicians can improve their ability to diagnose and manage TN effectively in sports medicine settings, ultimately enhancing athlete care and outcomes.

## References

[1] Gari SA (2013). A case of acquired trichorrhexis nodosa after applying new hair spray. J Saudi Soc Dermatol Dermatol Surg.

[2] Schwartz Schwartz, R. A., & Seiff, D. B. (2012 (2024). Trichorrhexis nodosa: practice essentials, pathophysiology, etiology. https://emedicine.medscape.com/article/1073664-overview?form=fpf.

[3] Whiting DA (1987). Structural abnormalities of the hair shaft. J Am Acad Dermatol.

[4] Miyamoto M, Tsuboi R, Oh-I T (2009). Case of acquired trichorrhexis nodosa: scanning electron microscopic observation. J Dermatol.

[5] Olsen EA (2003). Disorders of Hair Growth: Diagnosis and Treatment. https://books.google.co.in/books/about/Disorders_of_Hair_Growth.html?id=LvVsAAAAMAAJ&redir_esc=y.

[6] Itin PH, Fistarol SK (2005). Hair shaft abnormalities-clues to diagnosis and treatment. Dermatology.

[7] Shao L, Newell B (2014). Light microscopic hair abnormalities in children: retrospective review of 119 cases in a 10-year period. Pediatr Dev Pathol.

[8] McMichael AJ (2007). Hair breakage in normal and weathered hair: focus on the Black patient. J Investig Dermatol Symp Proc.

[9] Martin AM, Sugathan P (2011). Localised acquired trichorrhexis nodosa of the scalp hair induced by a specific comb and combing habit-a report of three cases. Int J Trichology.

[10] Taylor SC, Barbosa V, Burgess C, Heath C, McMichael AJ, Ogunleye T, Callender V (2017). Hair and scalp disorders in adult and pediatric patients with skin of color. Cutis.

[11] Lagrán ZM, González-Hermosa MR, Diaz-Pérez JL (2009). Localized trichorrhexis nodosa. Actas Dermosifiliogr.

[12] Fichtel JC, Richards JA, Davis LS (2007). Trichorrhexis nodosa secondary to argininosuccinicaciduria. Pediatr Dermatol.

[13] Burkhart CG, Burkhart CN (2007). Trichorrhexis nodosa revisited. Skinmed.

[14] Rudnicka L, Olszewska M, Rakowska A, Slowinska M (2011). Trichoscopy update 2011. J Dermatol Case Rep.

[15] Shah S, Ankad BS (2017). Trichoscopy of an isolated trichorrhexis nodosa: a case report. Indian Dermatol Online J.

